# Does neural computation feel like something?

**DOI:** 10.3389/fnins.2025.1511972

**Published:** 2025-05-23

**Authors:** Albert Gidon, Jaan Aru, Matthew E. Larkum

**Affiliations:** ^1^Institute of Biology, Humboldt University of Berlin, Berlin, Germany; ^2^Institute of Computer Science, University of Tartu, Tartu, Estonia; ^3^Neurocure Center for Excellence, Charité Universitätsmedizin Berlin, Berlin, Germany

**Keywords:** functionalism, computational functionalism, counterfactuals, counterfactual eraser, consciousness simulation, computer simulation

## Abstract

Artificial neural networks are becoming more advanced and human-like in detail and behavior. The notion that machines mimicking human brain computations might be conscious has recently caused growing unease. Here, we explored a common computational functionalist view, which holds that consciousness emerges when the right computations occur—whether in a machine or a biological brain. To test this view, we simulated a simple computation in an artificial subject’s “brain” and recorded each neuron’s activity when the subject was presented with a visual stimulus. We then replayed these recorded signals back into the same neurons, degrading the computation by effectively eliminating all alternative activity patterns that otherwise might have occurred (i.e., the counterfactuals). We identified a special case in which the replay did nothing to the subject’s ongoing brain activity—allowing it to evolve naturally in response to a stimulus—but still degraded the computation by erasing the counterfactuals. This paradoxical outcome points to a disconnect between ongoing neural activity and the underlying computational structure, which challenges the notion that consciousness arises from computation in artificial or biological brains.

## Introduction

Brains perform a variety of computations like sound localization (Ashida and Carr, 2011), spatial navigation (O’Keefe and Nadel, 1978), depth perception (Nityananda and Read, 2017), language processing (Jackendoff, 2003), and many more. These functions are supported by a hierarchy of computations, from simple (low-level) to complex (high-level) processes, each utilizing unique systemic and cellular mechanisms. Recent technological advancements in areas such as brain simulation (Markram et al., 2015; Wang et al., 2024), brain emulation (Cassidy et al., 2024; Kaiser et al., 2022; Yan et al., 2019; D’Agostino et al., 2024), and multi-modal large language models (Girdhar et al., 2019; Zhang et al., 2024), are striving to compute with complexity parallel to the human brain. These innovations not only transform neuroscience and artificial intelligence but also underscore an ongoing growth in computational power and sophistication that historically was unique to biological brains.

Computational functionalism maintains that mental states and processes, including consciousness, are defined by their functional roles (Putnam, 1980; Fodor, 1975; Butlin et al., 2023). According to this view, consciousness and other mental phenomena arise from computational processes regardless of their physical medium. Interestingly, conscious experience emerges only from certain levels of the brain’s computational hierarchy, while other levels remain nonconscious or subconscious (Koch and Tsuchiya, 2007; Dehaene et al., 2006; Dehaene et al., 2014). It remains unclear why some levels of computation feel like something while others do not.

The assumption of substrate independence built into computational functionalism means that any appropriate computational architecture should exhibit the same mental and experiential states. These computations can occur in biological brains or artificial systems, aligning with the principle of “multiple realizability” (Putnam, 1967; Colombo and Piccinini, 2023). This principle suggests that machines could become conscious if they perform the “right” computations.

The Global Neuronal Workspace Theory (GNWT; Dehaene and Changeux, 2011; Dehaene et al., 2014) is an example of a leading functional theory that explains consciousness as the result of specific patterns of neuronal computation and global information sharing in the brain. According to GNWT, consciousness emerges when information is “globally broadcasted” across a network of interconnected neurons, known as the global workspace. This process, termed “ignition,” occurs when neural representations reach a threshold of activation, leading to widespread neural synchronization, particularly in the prefrontal and parietal cortices. If, as argued by this theory, computation is sufficient for consciousness, then artificial systems that implement a global workspace could achieve consciousness comparable to biological systems (e.g., VanRullen and Kanai, 2021). This conclusion works for GNWT and, by the same measure, for other computational theories of consciousness not mentioned here (Aru et al., 2023). Nevertheless, it is worth noting that while many endorse this conclusion, not all functionalists necessarily accept it. For instance, Shevlin (2024) nomenclature identifies “rejectionism” and “stringent conservatism” as approaches that exclude non-human consciousness because non-human brains may lack the required functional organization.

In support of the functionalist perspective, Pylyshyn (1980) proposed an influential thought experiment in which neurons in a subject’s brain were gradually replaced with functionally identical microchips (see also Chalmers, 1995; Haugeland, 1980). Pylyshyn sought to preserve cause-and-effect relationships between neurons while eliminating the biological substrate. Since the original computational properties and interactions were preserved by definition, the brain’s functional properties, including consciousness, persisted. Recently, we proposed a thought experiment (Gidon et al., 2022) that extends and complements Pylyshyn’s concepts. Unlike Pylyshyn, our thought experiment aimed to preserve the activity and the biological substrate while eliminating cause-and-effect relationships between neurons. To achieve this, we initially recorded the precise activity (e.g., the intracellular membrane voltage) of all the neurons in the brain during a specific conscious experience and then replayed the recorded activity back to the same neurons. This thought experiment primarily questioned the hypothesis that some aspect of neuronal activity (e.g., action potentials) causes consciousness. Accepting that brain activity causes consciousness led to progressively challenging scenarios, culminating in the *reductio ad absurdum* that disconnected and scattered neurons might give rise to a conscious experience.

Given the importance of computational functionalism, we aimed to understand some of its unexplored consequences. Therefore, we examined whether computation can account for consciousness, irrespective of its implementation—be it in simulations, emulations, or biological brains. Using the NEURON simulation environment (Hines and Carnevale, 1997), we simulated an experiment in which a visual stimulus was presented to an artificial subject, and the activity in its (minimalistic) brain was recorded and then replayed using a (simulated) voltage clamp technique (Sharp et al., 1993). Starting from a computational functionalist standpoint, we hypothesized that the computation performed by the subject’s brain underlies conscious experience. We then explored how counterfactuals influence computation in the artificial subject’s brain and, consequently, its consciousness. We arrive at paradoxical results that underscore the gap between the biophysical aspects of brain dynamics and the computational accounts of consciousness.

## Methods

**Figure 1:**
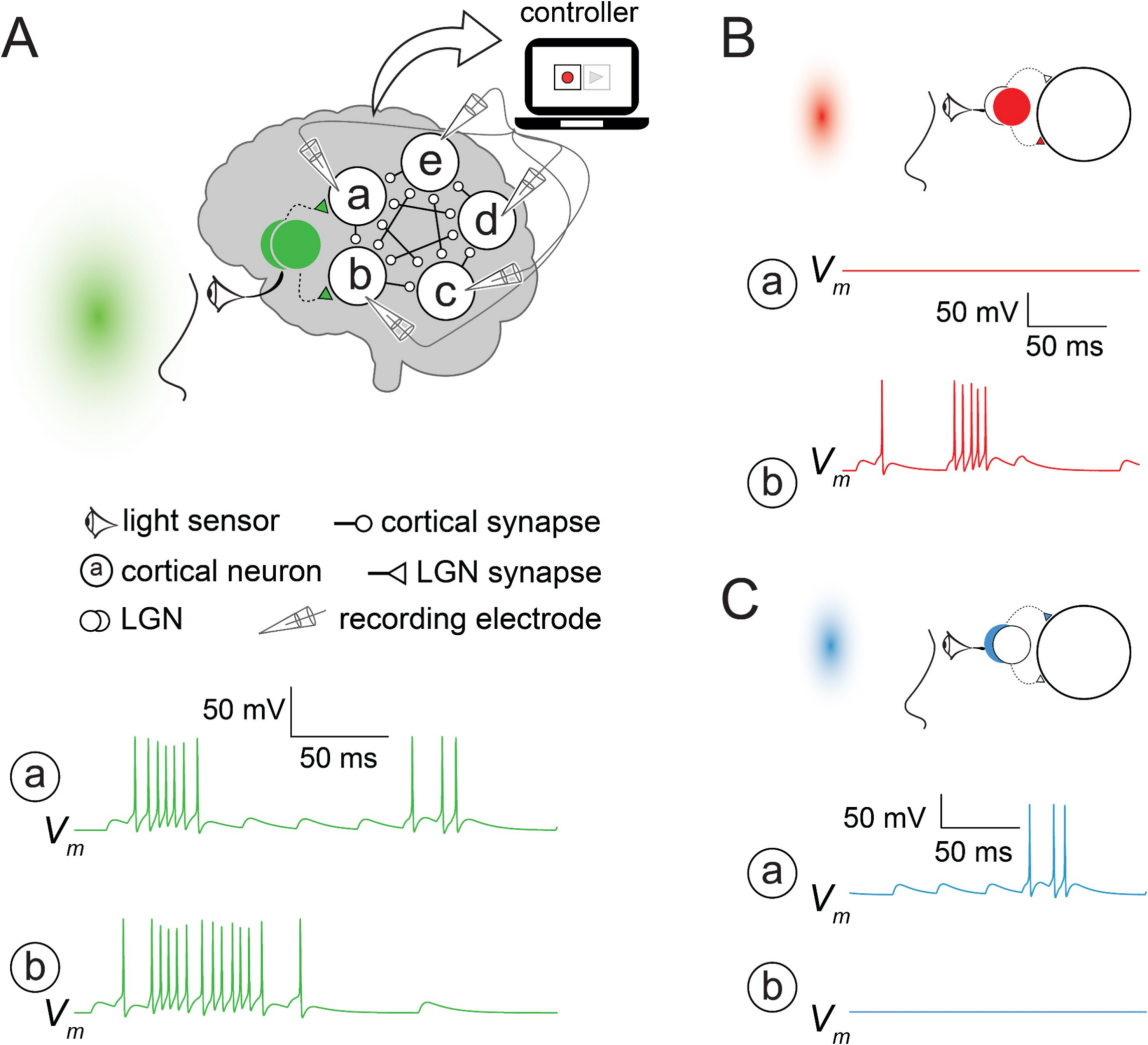
An artificial psychophysical experiment **(A)**. A simplified visual system modeled in NEURON simulation environment performs hue-computation. The eyes (sensor) detect the light and activate the two input nodes (lateral geniculate nucleus; LGN), which connect to neurons ***a*** and ***b***. When the green light turned on both LGN nodes, all cortical neurons (***a***–***e***) fired. Membrane voltage traces (*V_m_*) from each neuron were recorded for the green light stimulus and stored for later use by the replay. Traces are shown only for neurons ***a*** and ***b***. **(B,C)**. Red and blue lights activated only one of the LGN nodes, triggering action potentials in either neuron ***b*** (**B**) or ***a*** (**C**), which was insufficient to drive the other cortical neurons to fire.

The simulation was implemented using NEURON simulation environment (Hines and Carnevale, 1997); source code to reproduce all the traces for this study is available here in ModelDB (McDougal et al., 2017): http://modeldb.science/2018266. The visual cortex model included five identical neurons (circles in Figure 1, enclosing the letters *a–e*) and two input nodes (filled/empty overlapping circles in Figure 1). All neurons were assumed to be intrinsically inactive at the initial state or during the simulation unless they received synaptic input. Each cortical neuron was connected in cyclic order to the preceding three neurons; for example, neuron *d* received input from neurons *a*, *b*, and *c*. Each neuron was modeled by a single compartment with Hodgkin et al. (1952) biophysically inspired formalism (Traub et al., 1991) consisting of Na^+^, K^+^, and leak channels (*R_in_* = 50 MΩ). All synaptic inputs were modeled by a fast rise (2 ms) in the synaptic conductance and exponential decay (*τ_syn_* = 10 ms) triggered by presynaptic spikes. Synaptic conductances (*g_syn_* = 5 nS) were adjusted to drive the neurons to fire even though the number of synapses was small. To prevent runaway network excitation, we implemented short-term depression (resulting from the desensitization of synaptic receptors) for cortical synapses using first-order kinetics approximation (see equation 10 in Hennig, 2013). After each presynaptic action potential, the synaptic strength dropped to 50% of its value and recovered with a time constant of 1 s. Each LGN node was modeled as a random series of spikes that evoked synaptic potentials in their target cortical neurons. The eyes were modeled as a sensor that turned the LGN nodes on or off depending on the hue presented to the subject. Green light activated both LGN nodes, which drove the cortical network (Figure 1A) to fire. Other hues activated, at most, a single LGN, which was sufficient to drive only one cortical neuron above its firing threshold (Figures 1B,C). The built-in SEClamp object in NEURON was used to simulate the voltage clamp.

## Results

### Computation and consciousness

Similarly to our previous work (Gidon et al., 2022), we took the approach whereby it is sufficient to identify the target of the investigation rather than defining it precisely (Searle, 1998; see also Tononi, 2004; Tononi and Edelman, 1998). Hence, consciousness, as discussed here, refers to the experience of oneself or one’s surroundings that fades during deep sleep or anesthesia. Nonetheless, the simulation and conclusions in this work bypass the need for a precise definition, allowing the readers to rely on their own definition of consciousness.

The way we refer to computation aligns with the principles of a Turing Machine described by Turing (1936) and the principles of functionalism described by Putnam (1967). Specifically, computation is a process whereby a system receives various inputs that trigger state transitions according to a set of rules (or an algorithm), leading to an output. A key aspect for the functionalist is that the inputs, states, transitions, and outputs define the functional organization of the mind and are crucial to the conscious experience (Shagrir, 2005). A Turing Machine, according to this view, could experience pain and pleasure as long as it has the correct implementation of the tape (which stores its states) and the appropriate transition function (which guides the transition from one state to the next), whether in the form of a large language model, a neuromorphic chip, or even the human brain.

### The experiment

Inspired by a basic psychophysical experiment, we envisioned a scenario in which a subject is presented with a green light and instructed to press a button when she consciously perceives the light. We recorded the neuronal activity (i.e., the voltage) in every neuron of her brain immediately after the light turned on until she pressed the button. This time window captures the entire brain process underlying the conscious experience.

In the next step of the experiment, we played the recorded activity back into the same neurons (hereafter, the “replay”). Due to technical limitations and ethical considerations, such an experiment is currently not feasible in a large number of neurons in human subjects. Nevertheless, the replay provides an experimentally grounded conceptual framework that could, in principle, become feasible as technology advances. Indeed, it is evident that neuroscience has advanced in this direction (further discussed in Gidon et al., 2022).

Rather than relying entirely on a descriptive account of a thought experiment, we simulated it using a simplified model of an artificial subject’s brain. The simulation offers the reader more concrete results, while it requires minor conceptual adjustments when mapped to biological brains (see Discussion). The artificial subject consisted of a light sensor acting as eyes innervating two input nodes, each representing a lateral geniculate nucleus (LGN). The LGN inputs are then passed to the visual cortex, which is modeled as a recurrent network (for further details, see Figure 1 and the Methods section). For convenience, we referred to the response of the subject’s brain to different light hues as “hue-computation.”

**Figure 2:**
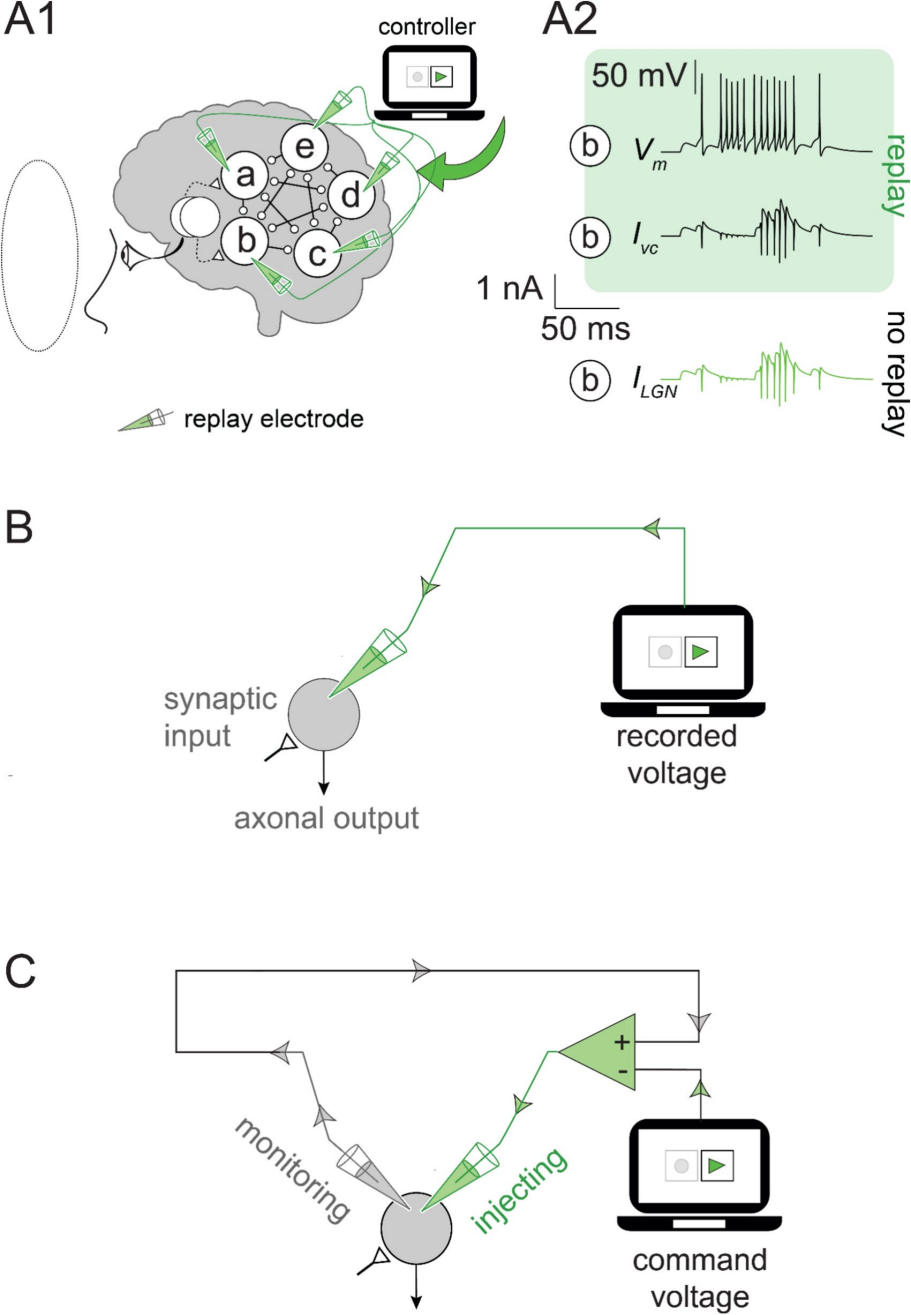
Feedforward and feedback replay mechanisms: **(A1)** The command voltage was set to the recorded voltage traces in Figure 1A (for the green stimulus) and replayed into the cortical network by a voltage-clamp amplifier. No visual input was presented to the subject (visual field indicated by the dotted line). **(A2)** Neuron ***b*** voltage (*V_m_*; *top*) was recreated by the voltage-clamp current (*I_vc_*; middle) during the replay without a visual stimulus. The current from the LGN input (*I_LGN_*; bottom) recorded under the green visual input without replay (as in Figure 1A) was identical to *I_vc_* during the green replay. **(B)** The feedforward replay: The neuron receives synaptic inputs, but they are ignored, and the neuron output is overwritten by the green electrode. **(C)** Feedback replay: The monitoring electrode (gray) measures the voltage. The command voltage (i.e., the recorded *V_m_*) is stored in the controller. The voltage clamp amplifier (green triangle) uses the difference between these signals to determine the current injected through the green electrode.

During the simulated experiment, as the green light was presented to the artificial subject (i.e., the “subject”), we recorded the voltage in all cortical neurons at each simulation time step. Then, we replayed the recorded activity into the same neurons (Figure 2). As expected, the subthreshold voltage and neuronal firing during the replay were identical to those observed in the recorded simulation (Figure 2A). The replay recreated the response of the subject’s brain, even in the absence of the stimulus, by precisely controlling the activity of each neuron.

This simulated experiment, although rudimentary, captured the conceptual framework used to study neural computation in biological and artificial brains and can be easily extended to more complex scenarios. If some readers believe that consciousness requires a larger network–perhaps one implementing a global workspace (Dehaene and Changeux, 2011) or higher—order computations (Brown et al., 2019; Butlin et al., 2023)—they are invited to substitute our simple computation with one of their own design. Furthermore, hue-computation is used here as a placeholder for a candidate computation associated with conscious experience.

### The hypothesis

Adopting the functionalist perspective, we start with a working hypothesis that some computations constitute conscious experiences. Adapted to our simulation, the hypothesis states that when a light stimulus is presented to our subject, its brain performs hue computation, thereby becoming hue-conscious. Initially, we accept this working hypothesis but shall reevaluate it later in light of the results. This hypothesis does not imply that a functionalist perspective equates every computation with consciousness or, specifically, hue-computation is necessarily conscious. To clarify this point further, we postulate that all computations are divided into core and auxiliary computations. By definition, altering a core computation’s input/output function impacts conscious experience. An auxiliary computation, in contrast, manages various brain functions with no direct effect on consciousness, even when its input/output function is modified. Analogously, the motor is a core mechanism that, when modified, affects the car’s mobility. In contrast, the window roll-down actuator, although motorized, is an auxiliary feature with no role in the car’s ability to move. Functionalist theories imply a distinction between core and auxiliary computations, but not formally. Here, we assumed that the hue-computation performed by the subject is a core computation. Nevertheless, even if the reader disagrees with this assumption, the same experiment and conclusion could be applied to a core computation suggested by the reader. Additionally, it is worth noting that the findings discussed in this study depend on the fact that the replay is capable of altering computation, rather than the specific details of how it does so.

### Feedforward versus feedback replay

To understand the simulation’s outcome, it is crucial to clarify the role of the replay and the specifics of its implementation. The first approach we considered involved a feedforward mechanism that ignored the ongoing neural activity and overwrote the voltage in each neuron with the recorded values (Figure 2B). The feedforward replay eliminated the impact of the neurons on each other and effectively severed the connection between the neurons. Although such a replay would work, we also considered an alternative approach for reasons that will be clarified later. We sought a feedback mechanism that monitors the ongoing neuronal activity and nudges it toward target values. The voltage clamp is a common experimental technique in neuroscience laboratories and a natural candidate for achieving a feedback replay mechanism (Cole, 1949; Cole, 1972). Conducting a voltage clamp experiment requires a “command voltage” initially dialed into the voltage clamp amplifier and serves as a prescription for neural activity. The amplifier (illustrated in Figure 2C) monitors the neuronal voltage at every moment and compares it to the command voltage. When neuronal activity deviates from the command voltage, a current is injected into the neuron (green electrode in Figure 2C) to nudge the voltage back to the prescribed (command) value. In practice, the feedback correction is instantaneous, orders of magnitude faster than the neuron’s characteristic time. Thus, the voltage-clamp amplifier prohibits deviations from the command voltage during the experiment but does not otherwise intervene. To grant similar capabilities to our feedback replay, we simulated it as an experimental voltage-clamp amplifier (hereafter, we use the terms voltage-clamp and feedback replay interchangeably). We set the command voltage for each cortical neuron to the recorded activity evoked by the green stimulus. This ensured that the activity during the replay matched the activity caused by the green visual stimulus.

Both the feedforward and the feedback replays accurately recreated the cortical activity. However, the difference becomes apparent when no visual stimulus is presented to the subject. The feedforward replay ignored the ongoing network activity and overwrote the voltage in each neuron with the recorded values. In contrast, the feedback replay achieved identical results without overwriting the neuronal activity (Figure 2A). Instead, it accurately recreated the missing LGN inputs (Figure 2A2) to the cortical neurons by nudging the neuronal activity in the cortical network toward the command voltage moment-by-moment.

**Figure 3:**
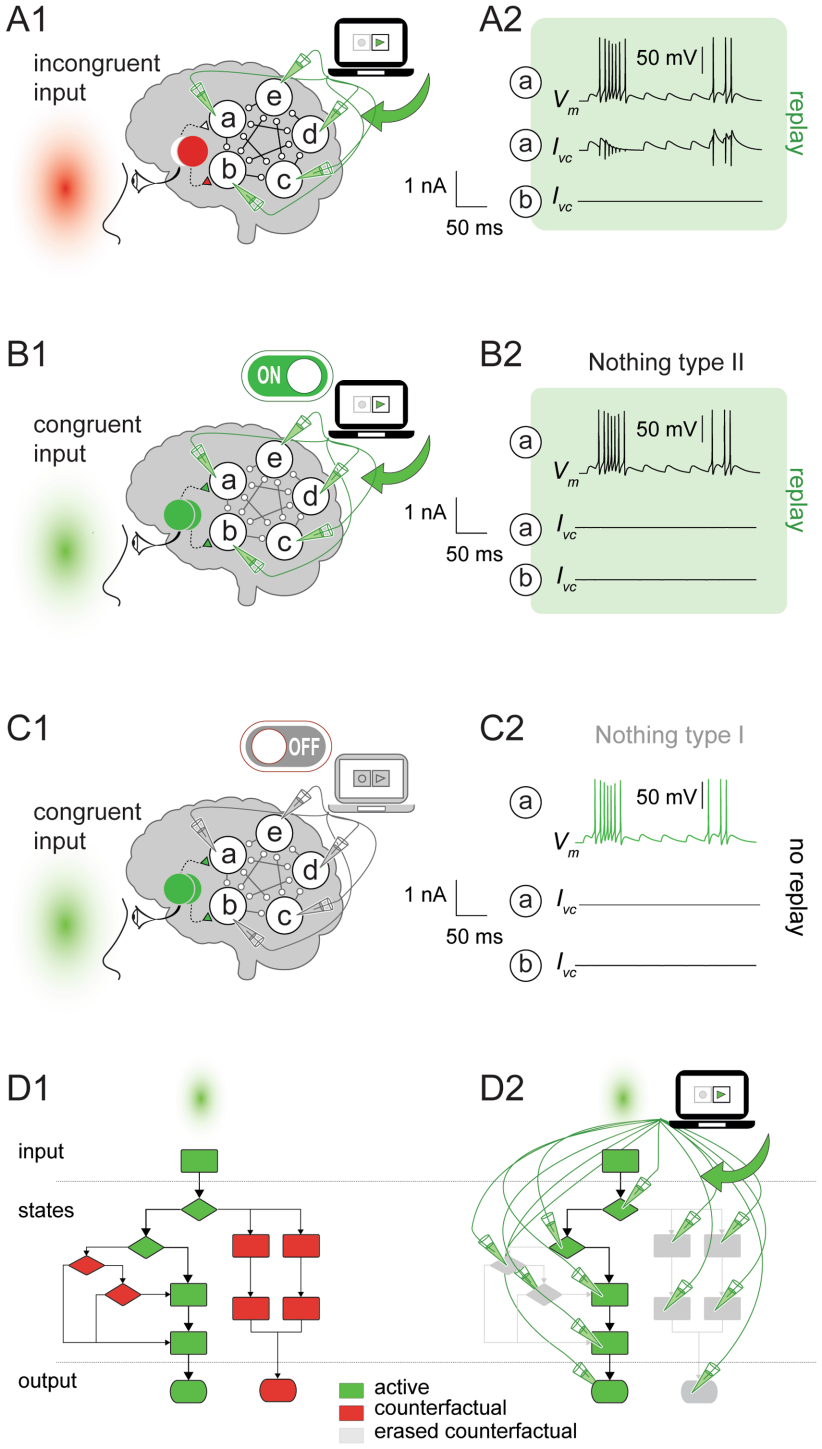
Paring the feedback replay with congruent and incongruent inputs. **(A1)** The command voltage was set to the recorded values for the green stimulus (see Figure 1A) and replayed into the cortical network by a voltage clamp amplifier during an incongruent stimulus (red light). **(A2)** *Top.* Voltage trace (*V_m_*) for neuron ***a***. *Middle.* The replay recreated the missing LGN input for neuron ***a*** (*I_vc_*). *Bottom*. Neuron ***b*** received input from the LGN, resulting in no replay currents (*I_vc_*). **(B1)** As in **(A1)**, but for the congruent input (green stimulus). **(B2)** *V_m_* (*top*) and *I_vc_* (*middle*) for neuron ***a*** during the replay. No currents were injected during the replay because neurons ***a*** and ***b*** received inputs from the corresponding LGN nodes (Nothing type II; *bottom*). **(C1,C2)** as in **(B1,B2)**, but the amplifier was turned off, and therefore, no currents were injected by the voltage clamp (Nothing type I). **(D)** Counterfactual eraser operation demonstrated using a toy algorithmic computation. **(D1)** Without the replay, the algorithm executed a particular trajectory for the green stimulus (depicted by green blocks and solid lines). The alternative operation sequences (depicted by red blocks and dashed lines) remain available. **(D2)** as in **(D1)**, but during the replay. The algorithm degenerated to one prescribed sequence of operations with no counterfactuals.

To further illustrate the distinct impact of the feedback replay, we introduced an incongruence between the stimulus and the replay. Namely, we presented a red light (that activates only one LGN node) while replaying network activity for the green light (activating both LGN nodes). As expected, during the replay, all the cortical neurons fire as if the green light was presented rather than the red light. Notably, the replay did not recreate (or overwrite) the entire network activity from scratch as in the feedforward case; instead, it factored in the ongoing network activity driven by one LGN node (i.e., for the red stimulus) and supplemented it with the missing input from the second LGN node (Figure 3A). The replay remained equally effective whether the neurons in the subject’s brain were disconnected or rewired, or a new input was introduced (not shown).

### The replay results in a degenerate computation

**Figure 4:**
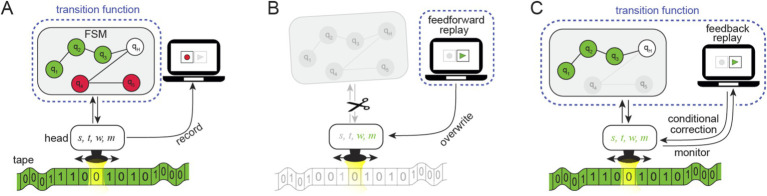
The replay and its impact on the elements of computation in a Turing Machine model: Reframing the simulated experiment as a Turing Machine consisting of a transition function given by a Finite State Machine (FSM), a moveable read/write head, and a tape. The head keeps track of the following parameters: the current and next states, reads and writes a symbol on the tape, and moves the head to the next position [parameters *s, t, w, m,* respectively]. **(A)** Recording: The Turing Machine operates based on a transition function given by the FSM and the tape. The green light presented to the subject is embedded in the green tape (in addition to other read/write data), and a sequence containing the states, symbols, and movements is recorded (i.e., recording *s, t, w, m*). **(B)** Feedforward replay: The replay instructs the head to write and move, overwriting *w, m* (in green) and ignoring *s*, *t* (grayed out) using the sequence of the recorded operations, bypassing the FSM. The new transition function consists only of the replay. **(C)** Feedback replay: The feedback replay monitors the reads and the writes and corrects them in cases where they deviate from the recorded sequences (due to changes made to the tape or the FSM). Given a congruent green input (embedded in the green tape), the replay only monitors, allowing the FSM to interact with the head. The new transition function includes the replay and the FSM (encircled by the dotted line) with erased (grayed-out) states. The outcome amounts to a trivial computation consisting of a single sequence of states (i.e., universally realizable).

The replay fixed the cortical network state transitions and outputs by effectively decoupling neurons from the visual input or any other influence that could alter their prescribed behavior. Given that replay modified the key elements of computation, namely, the inputs, state transitions, and outputs, we concluded that the replay altered the original hue computation or possibly even eliminated it. The impact of feedforward and feedback replay on computation is apparent in the diagram representing a toy algorithm (Figure 3D), where computational states and outputs are mapped onto the different diagram branches. The replay erased all the algorithm’s branches (in gray) except the recorded one (in green). Specifically, the replay pruned some states, eliminated their interactions, and overrode the algorithm rules, degenerating the computation into a fixed sequence of operations independent of the input. The degenerate computation represented a fundamental change in the *functional organization* of a system implementing the algorithm. From the functionalist’s perspective, changing the functional organization of a system is synonymous with changing consciousness (Putnam, 1980; Shagrir, 2005; Block, 1978; Lewis, 1972), which implies that replay altered the conscious experience (see Figure 4 and corresponding discussion).

### Consciousness and nothing

In the previous sections, we described the outcome of an incongruent stimulus presented during the replay (Figure 3A). Next, we introduced a congruent input by presenting the subject with a green light while replaying the previously recorded green activity (Figure 3B). During the replay, the brain activity for the congruent and the incongruent inputs was identical: Both led to a single set of state transitions irrespective of the input (compare Figures 3A,B). Therefore, computation degenerated for both cases during the replay. However, in contrast to the incongruent input (Figure 3A2), the voltage clamp did not inject any current into the neurons for the congruent input because no intervention was needed to obtain the prescribed behavior (Figure 3B2). Therefore, cause-and-effect relations between the neurons in the subject’s brain could naturally evolve, just as they would without the replay (here we rely on a mechanistic perspective on causation, e.g., Ross and Bassett, 2024, rather than on the counterfactual approach as presented by Lewis, 1973). This result demonstrates the paradoxical consequences of the congruent input: On the one hand, the replay altered consciousness by changing/degenerating computation. On the other hand, it did not alter brain activity and left the cause-and-effect relations between the neurons intact (i.e., it did nothing). If the replay did nothing, how could it change consciousness?

We distinguish two types of doing nothing: “Nothing type I” (Figure 3C) and “Nothing type II” (Figure 3B). Nothing type I describes the replay doing nothing simply because the voltage clamp amplifier was switched off. As follows from our working hypothesis, the subject was hue-conscious for Nothing type I. In contrast, “Nothing type II” describes the case where the voltage clamp amplifier was switched on but did nothing because the brain activity was identical to the command voltage. As described earlier, Nothing type II alters hue-computation and, according to our working hypothesis, hue-consciousness.

### Counterfactual erasers

The distinction between Nothing type I and Nothing type II is unrelated to the ongoing brain activity that follows the congruent input because it was identical for both Nothings. Instead, the difference depended on counterfactuals. i.e., the hypothetical activity (that did not happen) for other visual stimuli that were not presented and possibly will never be presented (Figure 3D). Therefore, we propose viewing the feedback replay as a “counterfactual eraser” because it can prohibit only counterfactuals without affecting anything else, i.e., brain activity and cause-and-effect between the neurons. The counterfactual eraser reveals a paradox: erasing abstract computational scenarios, which have no real-world impact on how brain activity responds to a specific (congruent) input, can nonetheless alter or even abolish consciousness.

Counterfactual erasers can also operate at the input level, for instance, by recording and replaying the subject’s LGN nodes activity while a green light is presented, or analogously, by having the subject wear ‘green glasses’ that convert all stimuli to green. Unlike the cortical replay, green glasses do not affect the computation underlying the green experience. From a functionalist perspective, although both may result in identical brain activity, only cortical replay affects the conscious experience.

## Discussion

In this work, we explored the relationship between computation and consciousness by simulating an artificial subject’s brain in response to visual stimuli of different hues. We started with a working hypothesis from a computational functionalist perspective whereby a subject performing hue-computation is hue-conscious. The ongoing activity in each neuron in the subject’s brain was recorded when a green light was shown and subsequently replayed into each neuron. To achieve that, we used a voltage clamp to inject current into the subject’s neurons, nudging them to the prescribed behavior obtained during the recording. We introduced the notion of counterfactual erasers (implemented by the replay) as an oversight device that continuously monitors the behavior of the system’s elements. This device intervenes only if one or more system elements deviate from the prescribed behavior. The key feature of counterfactual erasers is their *potential intervention* rather than their *actual intervention.* Therefore, the counterfactual eraser presents a unique situation whereby the interaction between the neurons and the activity of the network continued undisturbed even though all counterfactuals were erased. Consequently, counterfactual erasers can degenerate computations while doing nothing (Figure 3). If the reader accepts the working hypothesis, they must contend with the counterintuitive outcome that doing nothing to the brain can alter consciousness.

The importance of counterfactuals for computations underlying consciousness has been discussed before (Chalmers, 1996; Bishop, 2002; Maudlin, 1989; Klein, 2018), and therefore, we will briefly describe it here. Putnam (1988) and Searle (1990), among others, argued that computational sequences without counterfactuals are trivial and “universally realizable” thus, implemented by every system that transitions between states, even a rock (Chalmers, 1996). Accepting that trivial computations are sufficient for consciousness also undermines the conventional wisdom that complexity is a significant driver for conscious experience. Instead, according to this view, even simple machines performing simple computations could already be conscious.

### Explaining the replay within a Turing machine model

A Turing Machine (Turing, 1936) is a fundamental model of computation comprising an infinite tape, a read/write head, and a finite set of rules (a “transition function”) controlling its behavior. This model can simulate any computational process, making it a cornerstone of computer science and central to discussions about consciousness from a functionalist perspective.

To clarify how replay influences computation, we examine both the feedforward and feedback replay mechanisms within a Turing Machine framework. Our intention in this section was not to establish a strict mapping between components of the artificial subject’s brain and the Turing Machine, but instead to relate the replay mechanism to the broader concept of computation. Within the Turing Machine model, the input is embedded in the tape (e.g., a “green tape,” Figure 4A) rather than presented as light input to the subject (without implying a direct mapping between the LGN and the tape which contains other read/write data relevant to the computation). Typically, the transition function is governed by a finite state machine (FSM) that determines the head’s read/write actions based on the tape content, allowing the input-dependent information to be processed dynamically.

During feedforward replay, the FSM is effectively bypassed, the input is ignored, and the head strictly follows the replay’s prescribed instructions. These instructions are derived from a linear sequence of recorded writes and moves (corresponding to the parameters *w* and *m* in Figure 4B). This behavior is independent of the input, whether congruent (e.g., “green tape” matching the replayed sequence) or incongruent (e.g., “red tape” or any other tape that does not match the replayed sequence). The feedforward replay can be viewed as a new implementation of the transition function that does not read the tape and ignores the FSM. As a result, the new transition function leads to a degenerate computation and, arguably, even entirely abolishes it.

The feedback replay imposes the same sequence of write and move operations as the feedforward replay, and the resulting input/output function is identical in both cases. However, it constrains the Turing Machine differently. Rather than ignoring the FSM as in the feedforward case, the feedback replay *requires* the head to interact with the FSM. When the head’s operations deviate from the recorded sequence (Figure 4C), the replay overwrites them.

Constructing a computational framework forces us to explicitly place the feedback replay within the components of the Turing Machine (Figure 4C). At first glance, the feedback replay might seem ‘external,’ like the patch clamp device appears external to the brain in our psychophysical experiments. Moreover, the patch clamp device does not alter the brain’s molecular structure, physical makeup, or neuronal interactions. Therefore, as it is external to the brain, it may also appear external to the computation itself. This impression is enhanced by the congruent input, during which the replay remains inactive (Nothing Type II). It is not immediately obvious how an external device that does not intervene could be integrated as part of the computation performed by the Turing machine.

Rather than considering whether the replay is internal or external, the question asked by the funcitonalist is whether the replay changes the functional organization of a system. However, the fact that the functional organization *has* changed can be readily demonstrated by simply presenting different (incongruent) inputs during the feedback replay. States that would have been reached through the evolving read/write interplay are unreachable. This process effectively erases these states, similar to the feedforward case. This erasure is *effective* in a conceptual sense, even though they are not physically erased in the FSM. This leads to the conclusion that the feedback replay device is conceptually part of (and alters) the Turing Machine by directly altering the Transition Function (Figure 4C, dotted line).

The magnitude of the change to the transition function will depend on the proportion of states used in the congruent case compared to all other cases. In principle, the replay could have a considerable impact, raising the possibility that conscious experience associated with this computation might be severely diminished or even abolished. The Turing Machine analogy delineates the conceptual (or computational) role of the feedback versus feedforward replay scenarios.

### Implications for biological networks

Because our artificial subject’s brain was inspired by the biological neural network and relied on widespread experimental techniques, our conclusions translate more naturally to real neuronal systems. We can map the five neurons in our simulated network to an experiment involving five biological cultured neurons (Hales et al., 2010) and, subsequently, record and replay the neuronal activity in the cultured network. As a result, counterfactuals will be erased, and network computation will degenerate regardless of the substrate. A similar outcome is expected when replacing the artificial neurons in a transformer architecture with realistic model neurons [e.g., the neurons in the Blue Brain Project, Markram et al. (2015)] or even biologically cultured neurons. A replay experiment in the human brain is technically challenging, much more than in a neuronal culture, but conceptually, both are straightforward. However, one apparent difference between simulation and living tissue is that the latter consists of intrinsic stochasticity, which is fundamental to the biological structure and function at every level of detail. It is impossible to replay the noise at the molecular level of the biochemical reactions and other low-level quantum noise. However, the replay can deal with the noise’s functional consequences on the electrical behavior of each neuron, similar to any other recorded input. It would undo the ongoing noise and reintroduce the recorded noise.

Some theories of consciousness, using “counterfactual thinking” (Epstude and Roese, 2008) and the “Multiple Drafts Model” (Dennett, 1991; Dennett and Kinsbourne, 1995), leverage biological stochasticity for computing alternative scenarios or realities. Such computational theories of consciousness that do not require true stochasticity are vulnerable to the effects of the counterfactual eraser. However, for theories that require true stochasticity (e.g., Van Hateren, 2019; Hameroff and Penrose, 1996), the implications of the counterfactual eraser on consciousness are difficult to determine, and we will not address them here.

### Erasing the counterfactuals from the perspective of integrated information theory

Integrated Information Theory (IIT), as proposed by Tononi and colleagues (Albantakis et al., 2023; Tononi et al., 2016), offers a profoundly different view on consciousness than most existing theories. Although it has a computational flavor, appreciating the functional organization of a system (e.g., the brain), it emphasizes the system’s complex functional causal relations arising from the physical components. The internal perspective is manifested by the intrinsic cause-and-effect powers of the system on itself, as determined by its states and transitions’ repertoire. The Transition Probability Matrix (TPM) describes the transition probabilities between all pairs of possible present and future states within a system. IIT uses the TPM to evaluate the causal structure formed by the system’s components and their interactions, and computes the integrated information (*Φ*, ‘big Phi’), which can fluctuate from moment to moment (Albantakis et al., 2023). IIT does not attribute consciousness to the specific computations being performed at any given moment. Instead, it relies on the repertoire of potential states and transitions within a system, namely the counterfactuals. Therefore, from the perspective of IIT, the critical aspect of the feedback replay is its ability to erase counterfactuals. For IIT, whether the feedback replay allows brain activity to evolve naturally without interference is less important.

Due to its distinct “unit grain” (Oizumi et al., 2014; Albantakis et al., 2023; Marshall et al., 2024), the replay may operate as background constraints or even redefine the neurons’ behavior. However, because its features are distinct from neuronal mechanisms (by design), it should not be considered part of the neural network. For one, it responds much faster than neurons, and thus, it operates on a distinct temporal scale in the context of integrated information. Furthermore, it continuously reacts to small changes in membrane potential (like voltage-dependent ion channels) rather than an all-or-non response to action potentials typical to neurons. Consequently, the replay degenerates the TPM by restricting the system dynamics. The degenerate TPM results in a simpler cause-and-effect structure that dramatically reduces *Φ*. To understand this outcome, it is essential to differentiate between *cause-effect powers or structure* (Albantakis et al., 2023) and *actual causation* (Albantakis et al., 2019). According to IIT, a cause-effect structure unfolded from a substrate is necessary and sufficient to account for all features of consciousness. In contrast, the cascade of cause-and-effect events in the current moment, namely, the actual causation (Albantakis et al., 2019), is less relevant to *Φ.* For example, the brain can have high *Φ* even if all the neurons are silent and do not cause each other to fire, provided its cause-effect powers are intact. Inversely, consciousness is dramatically affected by eliminating the cause-and-effect structure, as done by the counterfactual eraser, even when all the neurons are active as before. In conclusion, we underline a fundamental counterintuitive aspect shared by IIT and computational functionalism. For both, the feedback replay can degrade consciousness, despite doing nothing (Nothing type II). However, counterfactual erasers do not present a formal challenge to IIT because, although they preserve what the system *does* (the focus of functionalism), they alter what the system *is* [the focus of IIT; see Tononi et al. (2025)].

### More on counterfactual erasers

Harry Frankfurt suggested a famous thought experiment (1969) exploring the principle of alternative possibilities (i.e., counterfactuals) using a conceptual tool similar to the counterfactual eraser. Briefly, the thought experiment goes as follows: John plans to commit an immoral act. A monitoring device is implanted in Jones’ brain, without his knowledge, to ensure he commits this act in case he decides to change his mind. Jones independently chooses to commit the act, so the device remains inactive. Is Jones morally responsible for the action despite the absence of alternate possibilities? Frankfurt concludes that voluntary behavior rather than counterfactuals determines moral responsibility.

Our counterfactual eraser and Frankfurt’s device monitor ongoing activity and intervene only when prescribed behavior is not met. Despite the conceptual similarity, there are meaningful differences due to the question each device tries to tackle; one asks whether computation causes consciousness, whereas the other explores moral responsibility. Accordingly, Frankfurt’s device eliminates alternative outcomes by setting a predetermined future goal for Jones. Either slightly biasing Jones’s brain by manipulating a handful of neurons or completely controlling low-level activity in all his neurons, Frankfurt’s device would do the job as long as the goal is achieved. In contrast, we are interested in the ongoing activity of each neuron as the prescribed activity, regardless of the high-level outcome.

Suppose we smuggle a counterfactual eraser into Frankfurt’s thought experiment and use it on Jones’s brain instead of the original device. In that case, we can create an interesting scenario that is different from what Frankfurt envisioned. Activating the counterfactual eraser (assuming that we have the prescribed behavior of all the neurons in Jones’s brain) guarantees that he performs the act. If Jones can perform the act voluntarily precisely as prescribed, then the counterfactual eraser would do nothing (as in our simulations), and Frankfurt would hold him responsible. However, considering the alternate possibilities (i.e., counterfactuals) as crucial for computation and, therefore, for consciousness (Maudlin, 1989; Barnes, 1991), we may need to accept that Jones may have acted voluntarily but with altered or possibly diminished conscious experience. Can the functionalist hold Jones responsible even if he may have lost his consciousness?

### The risk of misconsciousing machines

**Figure 5:**
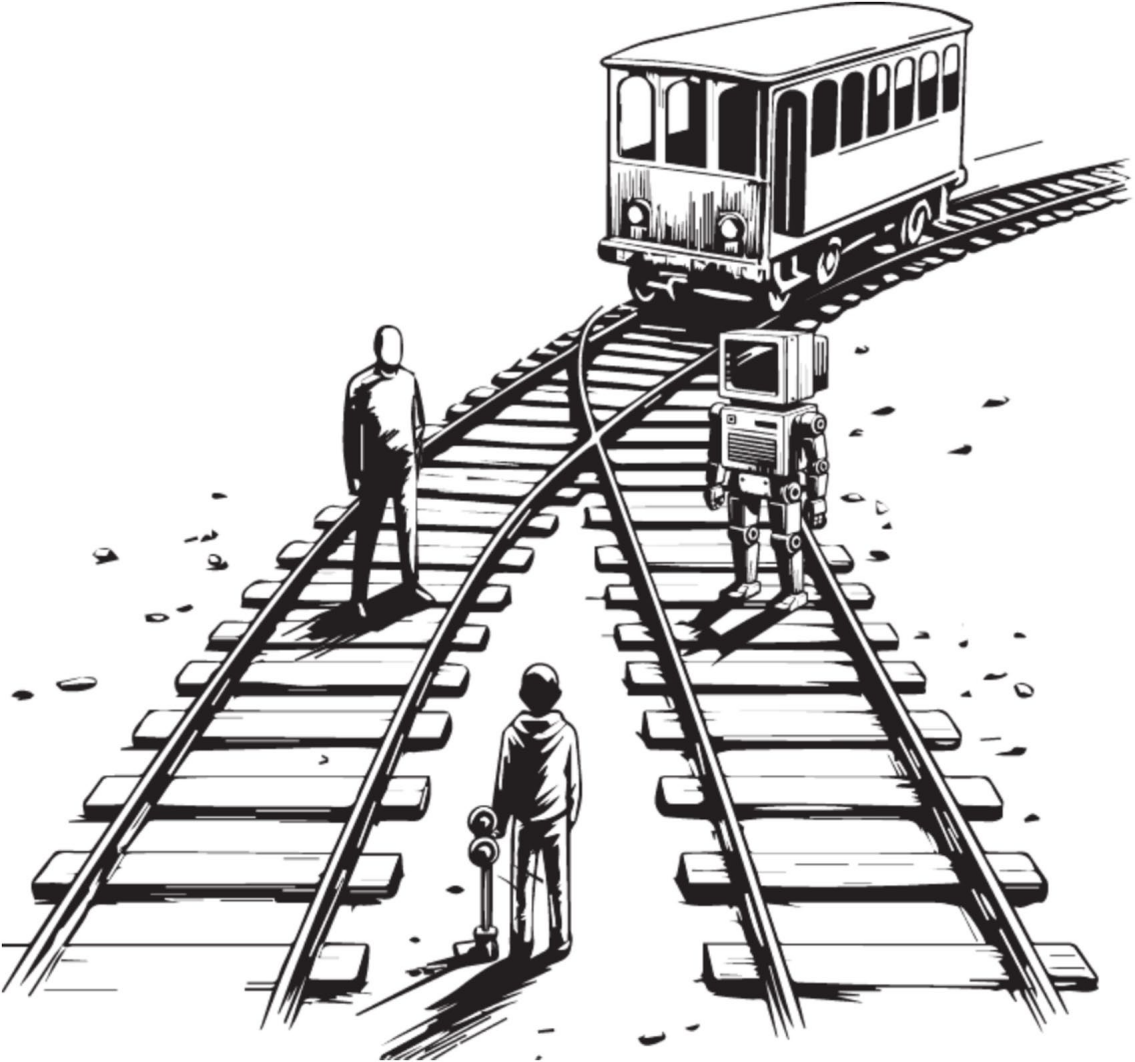
Functional computationalism promotes sudo-ethics. The classical moral dilemma (the ‘trolley problem’) introduced by Foot (1967) extended to an AI agent. Misconsciousing AI entails a risk that the perceived well-being of machines will come at the expense of humans (see Metzinger, 2013; Agarwal and Edelman, 2020).

Singer (1976) argued for extending some human rights to animals based on their ability to consciously perceive pain and pleasure. Most people view animals as conscious beings and animal rights protection is improving. In modern society, these rights are protected by legislation and, therefore, by law enforcement agencies. However, historically, philosophers often described animals as automatons and dismissed their expressions of emotion as reflexes (Thomas, 2020; Noble, 2023). This misconception has been largely corrected by a growing body of behavioral, neuroanatomical, and evolutionary evidence, which supports the common-sense view that animals share core capacities for consciousness with humans (Birch, 2020; Andrews et al., 2025; Irwin et al., 2022). However, the common sense that prevailed in the case of animals makes humans vulnerable to computer programs specifically designed to outsmart and (mis)use our intuitions (Shevlin, 2024; Colombatto and Fleming, 2024). The claim that AI might become conscious soon (or, according to some, may already be conscious) found its way to the mainstream (Finkel, 2023; Lenharo, 2023). Taking “mainstream assumptions, it’s a serious possibility that we’ll have conscious LLM + s within a decade” (Chalmers, 2024; see also yArcas, 2022; Shanahan, 2024). People more readily embrace views like these after engaging in human-like interactions with a chatbot. Whether or not machines have the capacity for conscious experience today or in the future has far-reaching implications. Just like animals, if machines are conscious, or even only possibly conscious, we are morally obligated to include them in our moral sphere. Some may feel obligated to prevent machine suffering and protect their rights, which may lead to corresponding legislation (Martínez and Winter, 2021; Shevlin, 2024). Misconsciousing machines could have significant societal repercussions, such as false ethical dilemmas (Figure 5) and skewed perceptions of machine-human relationships (Zhang et al., 2023). This problem could be exacerbated if instances of these machines are considered even more conscious than us; does your moral duty lie with your neighbor or with (what is claimed as) your hyper-conscious and best friend machine? As AI continues to advance and appears to blur the line between the conscious and the unconscious, we need better intellectual tools to clarify the difference between humans and machines (Findlay et al., 2025). Based on our work, we propose that the risk lies in ascribing machines with consciousness when they are not rather than the contrary.

### Does computation feel like something?

The concept of computation and its relationship to consciousness has been intensely debated in cognitive science and philosophy of mind. Views on computation vary widely; some scholars considering it a fundamental property of everything (Wolfram, 2002; Tegmark, 2015), particularly the mind (Fodor, 1975), while others dismiss it as merely an observer relative (Searle, 1980; Sayre, 1986) or a non-consequential interpretation of a physical process (Searle, 1990; Putnam, 1988). One of the core issues of the computation debate pertains to the relations between computation and the substrate realizing it (Colombo and Piccinini, 2023). While different substrates may compute similarly within a narrow range of conditions, their physical states can dramatically vary when examined over a broader range. For instance, temperature, pH, or pressure changes would affect biological substrates differently than a computer realizing the same computation. It is the substrate that determines the full range of the functional states–be it a human brain tissue or a microprocessor–rather than the computation we ascribe to it. Therefore, one may consider an alternative view that natural counterfactuals, rather than computational ones, are fundamental in determining our conscious experiences (see Deacon, 2013; Maudlin, 1989). Natural counterfactuals are embodied in the biological brain’s composition, structure, and dynamics and, therefore, cannot be erased without altering the brain’s physical structure and/or dynamics.

In conclusion, neural computation, which maps abstract algorithms to the brain’s dynamics, is valuable. When used carefully, it can be a powerful means of studying the brain, which is still the most sophisticated computer known to us. We better roll up our sleeves and explore the rich dynamics of the biological brain—the only known substrate capable of consciously experiencing the world.

## Data Availability

The code for the simulations presented in this study can be found in the following repository http://modeldb.science/2018266.
